# Purification and Identification of Peptides from Oyster (*Crassostrea hongkongensis*) Protein Enzymatic Hydrolysates and Their Anti-Skin Photoaging Effects on UVB-Irradiated HaCaT Cells

**DOI:** 10.3390/md20120749

**Published:** 2022-11-28

**Authors:** Zhilan Peng, Jialong Gao, Weimin Su, Wenhong Cao, Guoping Zhu, Xiaoming Qin, Chaohua Zhang, Yi Qi

**Affiliations:** 1 The Marine Biomedical Research Institute, Guangdong Medical University, Zhanjiang 524023, China; 2 College of Food Science & Technology, Guangdong Provincial Key Laboratory of Aquatic Product Processing and Safety, Guangdong Ocean University, Zhanjiang 524088, China; 3 The Marine Biomedical Research Institute of Guangdong Zhanjiang, Zhanjiang 524023, China; 4 National Research and Development Branch Center for Shellfish Processing, Zhanjiang 524088, China; 5 Guangdong Province Engineering Laboratory for Marine Biological Products, Zhanjiang 524088, China

**Keywords:** oyster, peptide, photoaging, purification, characterization

## Abstract

This study aimed to purify and identify antiphotoaging peptides from oyster (*Crassostrea hongkongensis*) protein enzymatic hydrolysates (OPEH) and to investigate the possible mechanism underlying its antiphotoaging effect. Multiple methods (Ultrafiltration, G25 Chromatography, RP-HPLC, and LC/MS/MS) had been used for this purpose, and eventually, two peptides, including WNLNP and RKNEVLGK, were identified. Particularly, WNLNP exerted remarkable antiphotoaging effect on the UVB-irradiated HaCaT photoaged cell model in a dose-dependent manner. WNLNP exerted its protective effect mainly through inhibiting ROS production, decreasing MMP-1 expression, but increasing extracellular pro-collagen I content. Furthermore, WNLNP downregulated p38, JNK, ERK, and p65 phosphorylation in the MAPK/NF-κB signaling pathway and attenuated bax over-expressions but reversed bcl-2 reduction in UVB- irradiated HaCaT cells. The molecular docking analysis showed that WNLNP forms five and seven hydrogen bonds with NF-κB (p65) and MMP-1, respectively. This study suggested that a pentapeptide WNLNP isolated from OPEH had great potential to prevent and regulate skin photoaging.

## 1. Introduction

The skin photoaging refers to a kind of cutaneous damage caused by the accumulation of exposure to ultraviolet (UV) lights [1], which is mainly characterized by brown spots, trans-epidermal water loss, epidermis hyperplasia, and degradation of collagen bundles in the dermis [2]. Compared to UVA (320–400 nm) and UVC (200–280 nm) lights, UVB (280–320 nm) exposure is the major contribution for photoaging progression [3]. Skin photoaging not only damages personal appearance but also has association with occurrence of many diseases, such as skin cancers [4,5,6]. How to prevent and regulate skin photoaging is one of the hotspots in the field of skin and cosmetics research.

Currently, various natural compounds [7,8], plant extracts [9,10], and protein hydrolysates [11,12] have been reported to exert protective effects on skin photoaging. Particularly, bioactive peptides from marine organs have gained more and more attention by researchers in recent years. Marine collagen peptides from deep sea fish [13], as well as peptides derived from tilapia gelatin [14] and cod skin [15], were reported to prevent skin from photoaging [15]. Hong Kong oyster (*Crassostrea hongkongensis*) is an economically important shellfish that largely cultivated in the shallow offshore waters of Southern China. Around 400 years ago, it had been recorded in “Compendium of Materia Medica” that the edibility of oyster is beneficial for smoothing and whitening skin. In addition, oyster and oyster-derived peptides have been discovered to possess multiple significant physiological activities, including antiapoptotic [16], antioxidant [17], anti-inflammatory [18], immunomodulatory [19], and protection against premature ovarian failure effects [20]. Pacific oyster hydrolysate was reported to have anti-melanogenic effects, as well as antiwrinkle formation, in UVB-irradiated mice skin [21,22]. A previous study from our group has also demonstrated that Oyster (*Crassostrea hongkongensis*) protein hydrolysates with low molecular weight protect against UVB-induced skin photodamage in Kunming mice [12]. However, the samples used for previous studies were still relatively complex, few purified compounds or peptides with antiphotoaging effect have been reported.

In this study, we aimed to isolate and purify bioactive peptide(s) with antiphotoaging effect from oyster protein enzymatic hydrolyte and then identify their amino acid sequences. Multiple methods (Ultrafiltration, G25 Chromatography, RP-HPLC, and LC/MS/MS) had been used for this purpose, and eventually, two peptides were identified with antiphotoaging effect. The protective effects of samples on UVB-irradiated keratinocytes HaCaT cells were investigated by CCK-8 kits. MMP-1 inhibition on UVB-irradiated HaCaT cells was also used for the screening of the antiphotoaging effect. Then, the mechanism underlying the antiphotoaging of identified peptide was further studied by Western blotting and molecular docking methods. The results from this study may provide reliable evidence for the application of peptides from oyster protein enzymatic hydrolyte as therapeutic or cosmetic products for treating skin photoaging.

## 2. Results

### 2.1. Hongkong Oyster (Crassostrea hongkongensis) Protein Hydrolysates (OPH) and Its Ultrafiltration Components Protected against UVB-Induced Damage in HaCaT Cells

**Figure 1:**
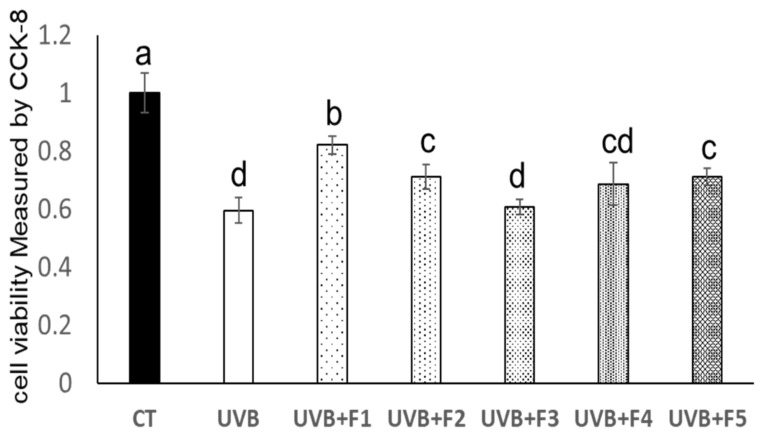
The effects of OPH and its four ultra-filtration fractions on UVB-irradiated HaCaT cell viability. F1, the fraction penetrated through 3 kDa membrane; F2, the fraction penetrated through the 5 kDa membrane but unfiltered through the 3 kDa; F3, the fraction penetrated through the 8 kDa membrane but unfiltered through the 5 kDa; F4, the fraction unfiltered through the 8 kDa filter membrane. F5, Hongkong Oyster (*Crassostrea hongkongensis*) protein Hydrolysates (OPH). The sample concentration of five fractions for the cell viability assay were 100 μg/mL, respectively. Different letters indicated significant differences between groups (*p* < 0.05).

Ultrafiltration is an effective purification method to prepare different molecular peptides from enzymatic hydrolysate [18]. In this study, the fraction with the lowest molecular weight (F1) showed the best antiphotoaging effect at the concentration of 100 μg/mL, which improved the HaCaT cells viability from 61.49% after UVB irradiation to 84.99% (*p* < 0.01) (Figure 1). In addition, previously, we have reported that this fraction exerted photoprotective effect on the skin of UVB-irradiated mice via inhibiting metalloproteinase 1 (MMP1) activity and its antioxidant activity. To confirm the components of this fraction, the F1 fraction was selected for further purification and identification analysis.

### 2.2. Peptides Separation by Sephadex G-25 Column

**Figure 2:**
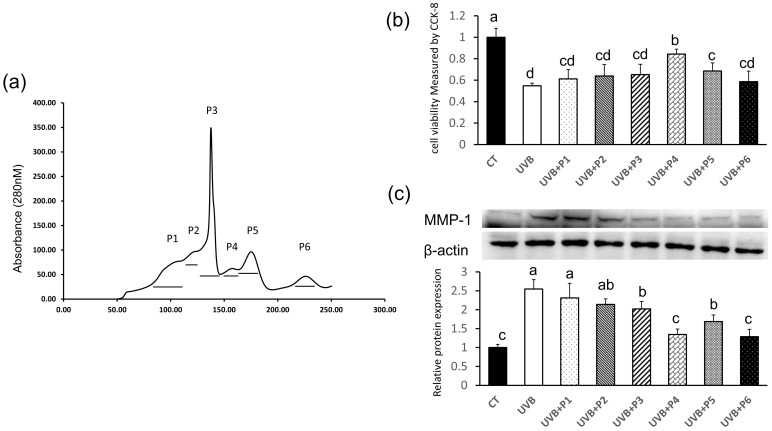
Separation and purification of peptides with antiphotoaging effect from F1. (**a**) The chromatogram of fraction F1 separated by Sephadex G-25 column, chromatography of F1 yielded 6 major peaks (P1–P6). (**b**) The antiphotodamage effects of six individual peaks on UVB- irradiated HaCaT cells. The sample concentration of six peaks for cell viability assay was 50 μg/mL, respectively. (**c**) The MMP-1 inhibitory activity of six individual peaks separated from F1. Different letters indicated significant differences between groups (*p* < 0.05).

After then, the F1 fraction was further isolated by a Sephadex G-25 column that could separate peptides below 5 kDa. Six subfractions (P1–P6) were collected in this step (Figure 2a), and their antiphotoaging effects were analyzed by testing cell viability of UVB-irradiated HaCaT cells (Figure 2b). As shown in Figure 2b, cell viability was significantly decreased after UVB irradiation (*p* < 0.01). However, among all subfractions, P4 subfraction showed the best effect of promoting cells from UVB-irradiated decrease in cell viability (*p* < 0.01).

Excessive degradation of extracellular matrix (ECM) by UV-induced MMPs is the important feature of photodamaged skin [23]. Particularly, MMP-1 serves as the primary MMPs in UVB-exposed skin, which is directly involved in the degradation of type I collagen, the major component of ECM in the dermis [24]. MMP-1 expression in HaCaT cells was also used as a major marker of photoaging in UVB-irradiated HaCaT cells [25]. Thus, MMP-1 expression was also measured in the presence of these six fractions after UVB irradiation in this study. As shown in Figure 2c, the expression of MMP-1 was significantly increased by UVB irradiation (*p* < 0.01). Although four fractions were found to inhibit UVB-induced MMP-1 over-expression, P4 exerted the best MMP-1 inhibition activity. Therefore, P4 was chosen for further purification (*p* < 0.01).

### 2.3. Peptides Separation by RP-HPLC

**Figure 3:**
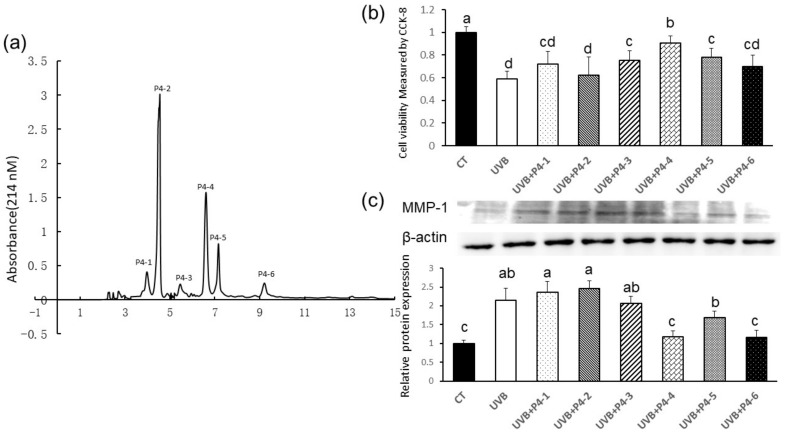
Elution curve of the fractions separated by reverse phase high performance liquid chromatography (RP-HPLC) and their individual effects on UVB-irradiated HaCat cells. (**a**) RP-HPLC chromatography of P4 yielded 6 major fractions (P4-1–P4-6). (**b**) The protective activity of individual fractions against UVB-induced damage on HaCaT cells. The sample concentrations for cell viability assay were 20 μg/mL, respectively. (**c**) The MMP-1 inhibitory activity of six individual fractions separated from P4. Collected fractions were concentrated and lyophilized for further study. Different letters indicate significant differences between groups (*p* < 0.01).

RP-HPLC is a reliable technique for analyzing peptides and proteins, which separates peptides based on its hydrophobicity property. Six major peaks were identified in the chromatogram of P4 (Figure 3a) and their antiphotoaging effects on UVB-irradiated HaCaT cells are shown in Figure 3b,c. As shown in Figure 3b, the peaks of P4-3, P4-4, and P4-4 shielded HaCaT cells from UVB-induced decrease in cell viability (*p* < 0.01 and *p* < 0.05, respectively). P4-4 was found to be the most potent fraction of all to protect HaCaT cells from UVB irradiation-induced cell viability decline (Figure 2b). Among the six fractions, P4-4 and P4-6 were two best peaks on inhibition of MMP-1 overexpression induced by UVB irradiation (*p* < 0.01, respectively). Considering P4-4 was better than P4-6 to protect HaCaT cells against a UVB induced decrease in cell viability (*p* < 0.05); therefore, the biological activity and sequences of peptides in P4-4 were further analyzed.

### 2.4. Peptides Identification by Mass Spectrometry

**Figure 4:**
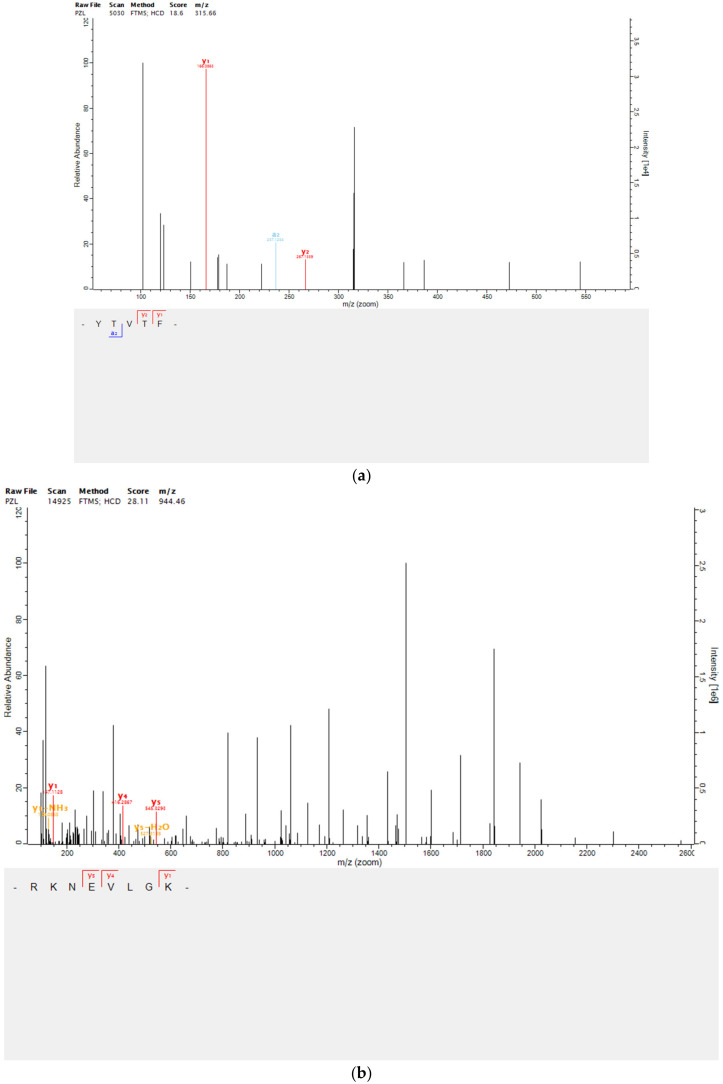

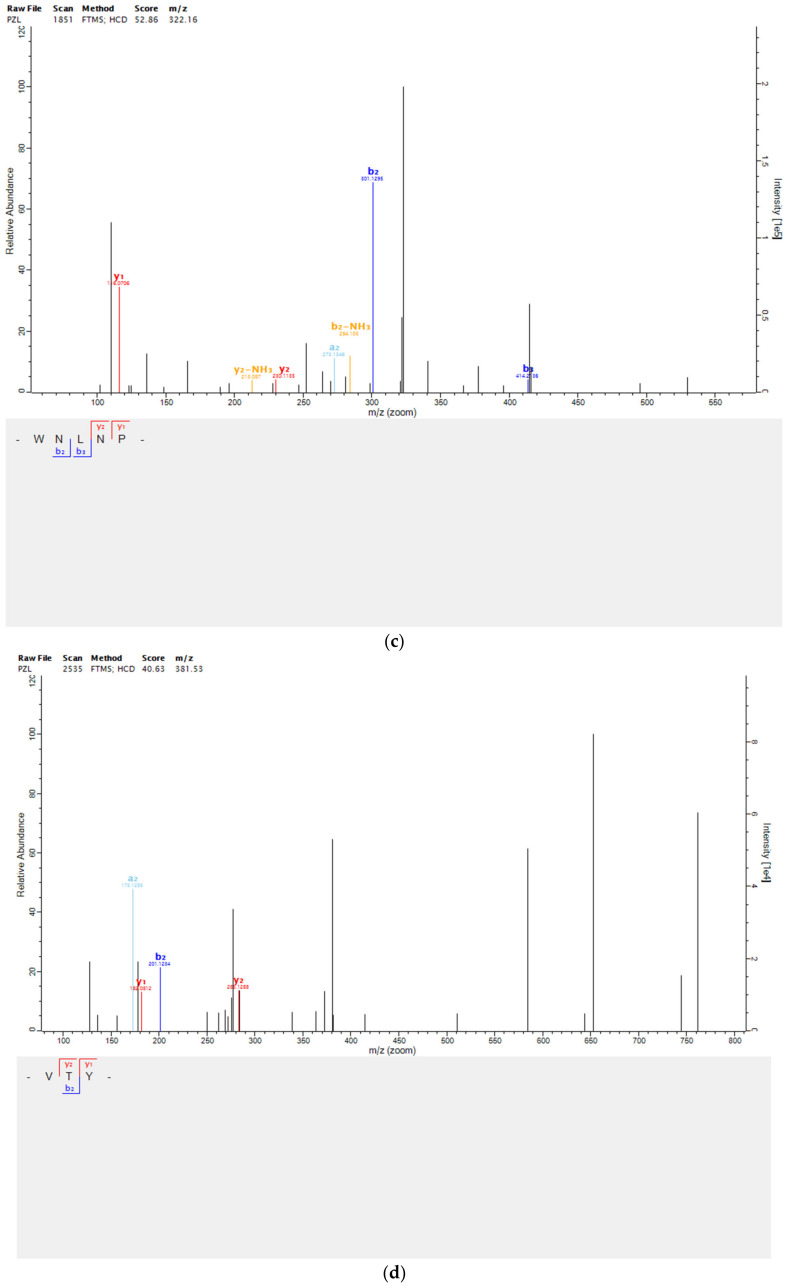
Peptide identification from the purified component P4-4 by UPLC-MS/MS. (**a**) Amino acid sequence of the peptide YTVTF, and ESI-MS/MS/MS spectrum of ion 315.66. (**b**) Amino acid sequence of peptide RKNEVLGK, and ESI-MS/MS spectrum of ion 944.46. (**c**) Amino acid sequence of the peptide WNLNP, and ESI-MS/MS spectrum of ion 322.16. (**d**) Amino acid sequence of peptide VTY, and ESI-MS/MS spectrum of ion 381.52.

For identifying the sequence of peptides, we purified the active fraction P4-4 by UPLC and further identified its structure by ESI-MS/MS. Totally, four peptides with higher confidence levels were selected and identified, and the MS/MS spectral of these four peptides are shown in Figure 4. These four peptides were identified as Tyr-Thr-Val-Thr-Phe (YTVTF), Arg-Lys-Asn-Glu-Val-Leu-Gly-Lys (RKNEVLGK), Trp-Asn-Leu-Asn-Pro (WNLNP), and Val-Thr-Tyr (VTY), and their theoretical molecular weights were 629.30 Da, 942.56 Da, 642.31 Da, and 381.18 Da, respectively.

### 2.5. Validation of the Antiphotoaging Effects of Four Peptides

Since few natural peptides could be separated from P4-4, these four identified peptides were artificially synthesized according to their sequence. Their antiphotoaging activity was measured by testing cell viability and MMP-1 inhibition ability on UVB-irradiated HaCaT cells, as mentioned before.

**Figure 5:**
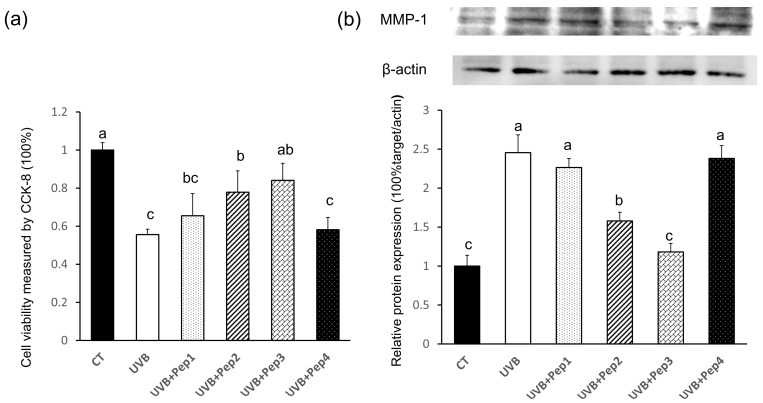
The protective activity of the four synthesized peptides on UVB-irradiated HaCaT cells. (**a**) The protective activity of synthesized peptides on cell viability of UVB-irradiated HaCaT cells. The sample concentration for cell viability assay was 20 μM. (**b**) The effect of synthesized peptides on the MMP-1 expression of UVB-irradiated HaCaT cells. Different letters indicate significant differences between groups (*p* < 0.01).

The results are presented in Figure 5. RKNEVLGK and WNLNP efficiently protect cells from damage after UVB irradiation (*p* < 0.05 and *p* < 0.01, respectively), whereas no significant difference was seen between effects of RKNEVLGK and WNLNP. Compared to the control group, UVB exposure significantly increased the protein expression of MMP-1 in HaCaT cells (*p* < 0.01). When treating RKNEVLGK and WNLNP, the activity of MMP-1 was dramatically decreased (*p* < 0.01, respectively). However, the inhibition effect of WNLNP on MMP-1 expression was better than that of RKNEVLGK (*p* < 0.01).

### 2.6. Effect of WNLNP on Cell Viability, Oxidative Stress, and Pro-Collagen I Production

To evaluate the pharmaceutical effects of WNLNP in vitro, cell viability was determined by CCK-8 assays as before. Different concentrations of WNLNP (10, 50, 100 μM) were applicated to the HaCaT cells that exposed UVB radiation. As expected, WNLNP efficiently protect HaCaT cells from UVB-induced damage in a dose-dependent manner.

**Figure 6:**
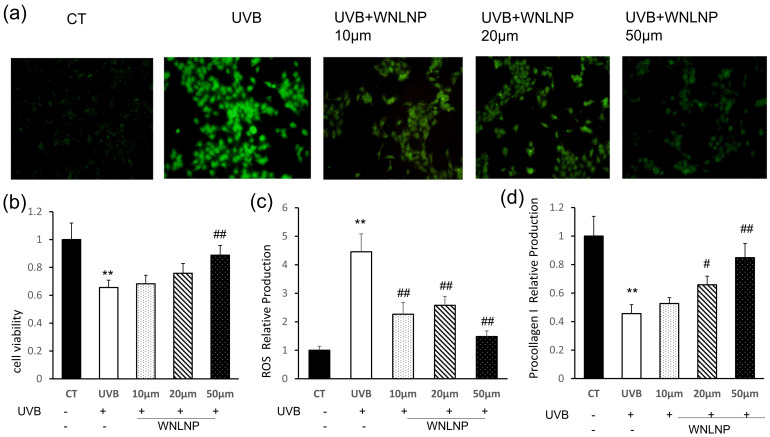
The protective effects of the WNLNP on UVB-irradiated HaCat cells. (**a**) DCF fluorescence of the treated cells was measured by using an inverted fluorescence microscope in UVB-induced HaCaT cells. (**b**) Effect of WNLNP on cell viability in UVB-irradiated HaCat cells. (**c**) The relative DCF fluorescence intensity of HaCat cells with different treatment. (**d**) Effect of WNLNP on pro-collagen I production. ** *p* < 0.01, compared with the CT group; # *p* < 0.05, and ## *p* < 0.01, compared with the UVB group.

As mentioned before, excessive UVB-induced ROS release leads to oxidative stress that damage cells [26,27]. Particularly, oxidative stress is known to be involved the progression of photoaging, and it is considered as a key trigger in the early stage of photoaging. To measure whether WNLNP could scavenge excessive ROS, HaCaT cells were treated with sample for 24 h after UVB irradiation, then ROS levels were detected by fluorescence probe. As Figure 6a,c shows, the ROS fluorescence was extremely promoted after UVB irradiation (Figure 6a,c). However, the UVB-induced increase in ROS fluorescence intensity was significantly declined following treatment with WNLNP in a dose-independent manner.

Collagen fibers, the main structural component of ECM, together with elastin fibers give the skin its strength and elasticity. Degradation of collagen is the most prominent histological character of photoaging [28]. However, pro-collagen is essential for the synthesis and formation of collagen. The ELISA results demonstrated that UVB irradiation dramatically declined the release of pro-collagen I in HaCaT cells. In contract, WNLNP (10, 50, and 100 μM) treatment following UVB exposure increased the production of extracellular pro-collagen I in a dose-independent manner (Figure 6d).

### 2.7. Regulated Effect of WNLNP on MAPK and NF-κB Signaling Pathways

**Figure 7:**
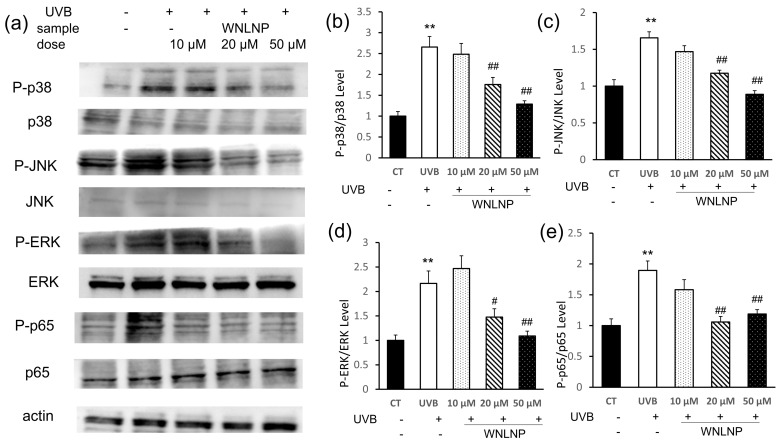
The effects of WNLNP on the phosphorylation of MAPKs signaling proteins (p-JNK, p-p38, and p-ERK,) and the NF-κB (p65) signaling pathway in HaCaT cells with different treatments. (**a**) Representative Western blot images of MAPK (p-JNK, p-p38, and p-ERK,) and NF-κB (p65) signal intensities in HaCaT cells from three independent experiments. (**b**) WNLNP mitigated the phosphorylation level of p38 in photodamaged HaCaT cells. (**c**) WNLNP mitigated the phosphorylation level of JNK in photodamaged HaCaT cells. (**d**) WNLNP mitigated the phosphorylation level of ERK in photodamaged HaCaT cells. (**e**) WNLNP mitigated the phosphorylation level of p65 in photodamaged HaCaT cells. All data are presented as the mean ± SD of three independent experiments, respectively, ** *p* < 0.01, compared with the CT group; # *p* < 0.05 and ## *p* < 0.01 compared with the UVB group.

To determine the effect of WNLNP on MAPK and NF-κB signal pathway in UVB-irradiated HaCaT cells, Western blotting analysis were conducted. As shown in Figure 7a–d, MAPK signaling proteins of ERK, JNK, and p38 in HaCaT cells were dramatically activated after UVB irradiation, which could be significantly downregulated by WNLNP administration in a dose-dependent manner.

The nuclear transcription factor-κB (NF-κB) plays essential roles in the cellular response to external stimulus, such as UVB radiation in the epidermis [29]. Inactive NF-κB subunits (such as p50 and p65) are present as a heterodimer in the cytoplasm, which combined to its inhibitor of κB (IκB) [30]. In the present of stimulating stimuli (including UVB irradiation), IkB degraded, and NF-κB subunits were transferred to the nucleus to regulate the expression of related genes [14,31,32]. As shown in Figure 7a,e, NF-κB (p65) was activated by the MAPK signaling pathway in UVB-irradiated HaCaT cells. However, NF-κB (p65) activation could be significantly downregulated by WNLNP treatment in a dose-dependent manner.

### 2.8. WNLNP Regulates the Overexpression of MMP-1 and Apoptosis-Related Signaling Pathway

**Figure 8:**
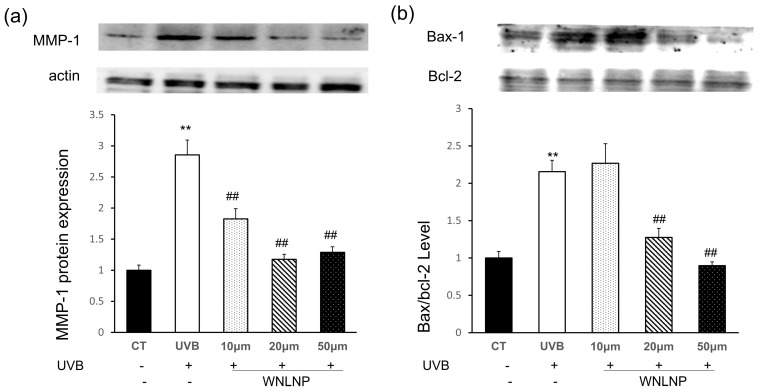
The effect of WNLNP on the protein expressions of MMP-1, Bax and bcl-2in HaCaT cells by Western blotting. (**a**): WNLNP mitigated the MMP-1 over-expression in photodamaged HaCaT cells; (**b**): WNLNP mitigated the apoptosis in photodamaged HaCaT cells All data are presented as the mean ± SD of three independent experiments respectively, ** *p* < 0.01, compared with the CT group; ## *p* < 0.01 compared with the UVB group.

Oxidant stress and inflammation response are activated upon UVB irradiation, which trigger signaling pathway cascades, followed by the elevation of MMP expression and consequent ECM degradation in the dermis. Upregulation of MMPs by UVB exposure is considered as an important factor that induces the apoptosis of keratinocytes in the literatures [14,23,33,34]. As mentioned before, MMP-1 is the primary MMPs in UVB-exposed skin. Thus, the inhibition of MMP1 (skin aging marker) is an effective attempt to reduce UVB-damaged apoptosis and the degeneration of ECM. In consistent with previous studies, the expression of MMP-1 was significantly increased after UVB exposure. However, WNLNP treatment declined the UVB induced-overexpression of MMP-1 in a dose-independent manner (Figure 8a).

In this study, the effects of WNLNP on the protein expression of proapoptotic and antiapoptotic genes were also tested by WB analysis. In according with previous studies [12,35], UVB irradiation significantly increased the expression of proapoptotic protein bax while decreased the production of antiapoptotic factor bcl-2 (Figure 8b). However, WNLNP administration after UVB irradiation could reverse the increase in bax expression and decrease the bcl-2 expression in UVB-irradiated HaCaT cells (Figure 8b). The current study may indicate that WNLNP inhibits UVB-induced apoptosis in HaCaT cells.

### 2.9. Molecular Docking Analysis of MMP-1 with WNLNP

**Figure 9:**
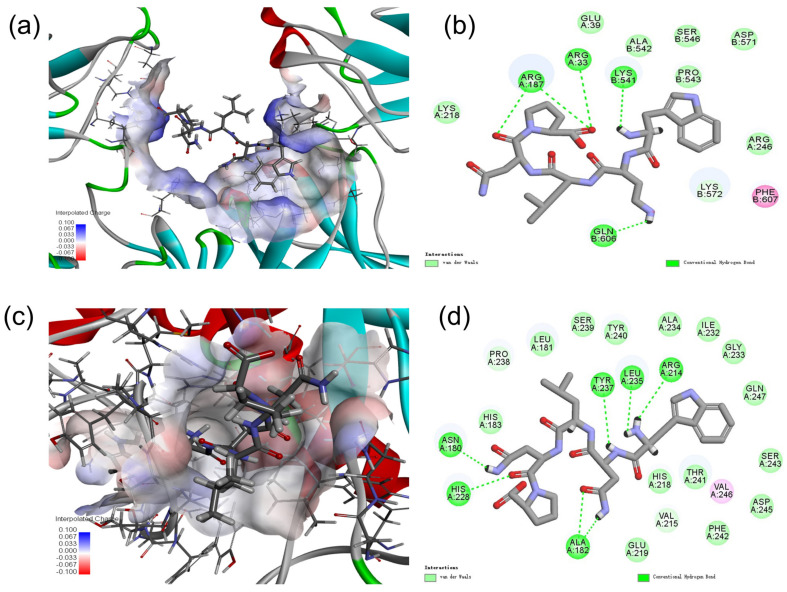
Molecular docking analysis of NF-κB(p65), and MMP-1 with WNLNP. (**a**) Optimal docking hydrogen bond 3D structure diagram of WNLNP and NF-κB(p65). (**b**) 2D diagram of the interaction between YGDEY and amino acid residues of NF-κB(p65). (**c**) Optimal docking hydrogen bond 3D structure diagram of WNLNP and MMP-1. (**d**) 2D diagram of the interaction between YGDEY and amino acid residues of MMP-1.

Molecular docking is an effective way to investigate the interaction between small molecule drugs and their targets. It has become a useful method for computer-aided drug research [14]. As shown in Figure 9. WNLNP have five hydrogen bonds residue with NF-κB (p65) (the residues of Arg33, Arg187 (two hydrogen bonds observed in residue of Arg187), Lys541 and Gln 606), formed shortest hydrogen bond at Lys541 with the distance of 1.80 A (Figure 9a,b). However, WNLNP have seven hydrogen bonds residue with MMP-1, formed shortest hydrogen bond at Asn180 with the distance of 2.13 A (Figure 9b,d). WNLNP strongly interacts with MMP-1, hydrogen bonds are observed between residues of WNLNP and the Asn180, Ala 182 (two hydrogen bonds observed in residue of Ala 182), Arg214, His228, Leu235 and Tyr 237 residues of MMP-1 (Figure 9b,d). Further docking analysis revealed that the interaction energy of peptide WNLNP with NF-κB (p65) with was −56.62 kcal/mol, while the interaction energy between MMP-1 and WNLNP was −68.83 kcal/mol.

## 3. Discussion

Previous study from our group have demonstrated that an ultrafiltration fraction of oyster (*Crassostrea hongkongensis*) protein hydrolysates with low molecular weight (F1 fraction in this study) exerts robust anti-skin photoaging effect in vivo [12]. However, exact peptides with antiphotoaging effect still need further purification and identification. In this study, four peptides were separated through a series of chromatographic methods. Eventually two peptides including WNLNP, RKNEVLGK were identified with antiphotoaging effects, and the effect of WNLNP was better than that of RKNEVLGK. Since WNLNP was the most effective peptide, it was chosen for studying the inhibition pattern and the possible mechanism underlying its antiphotoaging effect.

Keratinocytes are the outermost layer of the skin, which contains 95% of the cells in the epidermis. UVB exposure activates oxidative stress in keratinocytes [36]. In this study, the endogenous ROS level was significantly promoted by UVB irradiation, which was in line with previous studies that UVB exposure could decreases the activities of antioxidant enzymes, leading to irreversible ROS accumulation in HaCaT cells [14,37]. Increased ROS expression, on one hand, damages biological macromolecules such as DNA and RNA, eventually initiating key apoptotic events [38]. Previously, peptides from marine organisms such as *Isochrysis zhanjiangensis* [38], tilapia gelatin [14] and cod skin [15] were reported to prevent skin from photoaging by inhibiting ROS accumulation. In this study, WNLNP treatment decreased the endogenous ROS level while promoted its cell viability in UVB-irradiated HaCaT cells. To deduce its source of antioxidant activity, the composition of peptides with antiphotoaging effect was analyzed. Previous studies showed that peptides with molecular weights less than 1K Da and higher percentage of hydrophobic anomic acids are found to have better biological activity in term of protecting skin from photoaging [14,36,37]. A Peptide YGDEY (MW: 645.62 Da, hydrophobic aromatic residue Tyr at both the N- and C-termini) from Tilapia Gelatin Hydrolysates inhibited ROS over-expression by promoting antioxidant factors SOD and GSH contents in UVB-irradiated HaCaT cells [14]. In addition, Bang Joon Sok et al. isolated a peptide IVVFK (Ile-Val-Val-Pro-Lys) from oyster hydrolysate, which has three hydrophobic amino acids (60%) in the sequence [39]. Antiphotoaging peptide IVVFK recovered the activity of antioxidant enzymes and restored the amount of collagen in UVB-irradiated hairless mice [39]. In this study, WNLNP has three hydrophobic amino acids out of five amino acids, which significantly reduced ROS accumulation, as well as increased cell viability in UVB-irradiated HaCaT cells.

On the other hand, ROS could promote the expressions of pro-inflammatory cytokines and metalloproteinase (MMPs) [14,25], which eventually results in cellular senescence and the degeneration of extracellular matrix (ECM) [14]. Excessive degradation of extracellular matrix (ECM) by UV-induced MMPs is an important feature of photodamaged skin [12,23]. Particularly, MMP-1 serves as the primary MMPs in UVB-exposed skin, which is directly involved in the degradation of type I procollagen, the major component of ECM in the dermis [24]. MMP-1 expression in HaCaT cells was also used as a major marker of photoaging in UVB-irradiated HaCaT cells [25]. A variety of marine organism-derived peptides have been widely used to prevent photoaging by decreasing MMPs expression [13,14,36,37]. A peptide derived from chlorella suppressed MMP-1 expression, while increased procollagen mRNA level in human skin fibroblast irradiated with UVB [40]. Hydrolysates from cod skin gelatin exhibited antiphotoaging effects through inhibiting MMP-1 expression in UVB-induced mice fibroblasts model, and two peptides (GEIGPSGGRGKPGKDGDAGPK and GFSGLDGAKGD) were identified [41]. Thus, Decreasing MMP-1 expression to increase the type I procollagen production is considered as a promising strategy for skin photoaging therapies. In this study, WNLNP showed strongest MMP-1 inhibitory effect than other peptides. Further study revealed the most effective identified peptide WNLNP inhibited MMP-1 expression, while increase the production of pro-collagen I in UVB-irradiated HaCaT cells.

As crucial signaling pathways for the regulation of MMPs, MAPK and NF-κB could be activated by ROS over-expression. Considerable studies including ours [12,22,34,35,40] had revealed that MAPK could be activated through phosphorylation and activate downstream related pathways such as NF-κB. Extracellular signal-regulated kinases (ERKs), c-Jun N-terminal kinases (JNKs) and p38 are three dominant number of MAPK family, which engaged in UVB induced skin aging [38,42]. Upon UVB irradiation, Mitogen-activated protein kinase (MAPK) cascade pathways can transport extracellular signals into nucleus, activate downstream related pathway NF-κB [8,34,43]. MAPK and NF-κB together regulate the expression of cellular inflammatory factors and promote the generation of MMP-1 and MMP-9 [44,45]. Besides promoting the expression of MMPs, the activation of NF-κB signaling pathway induced by UVB irradiation subsequently promotes the expression of proapoptotic genes like Bax, Bad, and cleaved caspase-3, whereas simultaneously decreasing the production of antiapoptotic proteins such as Bcl-2 [46].Western blotting analysis in our study revealed that WNLNP significantly inhibited the p38, JNK phosphorylation in the MAPK signaling pathway, then limited the formation of p-p65 and p-IκB. In addition, WNLNP significantly inhibited proapoptotic Bax, but promoted antiapoptotic proteins expression Bcl-2 in UVB-irradiated HaCaT cells.

Previous studies including our group showed that the production of excessive ROS could activate NF-κB signaling pathway and affect the levels of MMPs [12,14,37]. Thus, the inhibitory effect of WNLNP on NF-κB signaling pathway and MMP-1 was possibly due to its scavenging ROS ability. It is still with great significance to determine whether peptide WNLNP has any direct interactions in the active sites of NF-κB and MMP-1 proteins to directly suppress their activities. Thus, the active-site residues of MMP-1 and NF-κB p-65 protein models were kept flexible and then docked with the WNLNP ligand. In the best molecular interaction pose, WNLNP formed five hydrogen bonds with NF-κB (p65). Except that, interaction energy between peptide WNLNP with NF-κB (p65) was with high negative values (−56.62 kcal/mol), indicating strong interactions between them. It’s well known that inactive NF-κB subunits (such as p50 and p65) are present as a heterodimer in the cy-toplasm, which combined to its inhibitor in normal condition [30]. Upon stimulation, inhibitor of NF-κB, could be degraded, then NF-κB subunit p65 was activated and transferred to the nucleus to regulate the expression of related genes [14,31,32]. The strong interaction between WNLNP and NF-κB was of significance for inhibiting the translation of p65 to the nucleus. This study clearly indicates that WNLNP might be a good inhibitor of NF-κB. It has been reported by several literatures that Hydrogen bond plays a significant role in stabilizing the crystallographic structures of complexes of enzymes and ligands [14,38,46]. Previous study showed that Leu 235 and Tyr 237 were key hydrophobic and polar amino acids of the S1 pocket of MMP-1 [37]. After docking, the peptide WNLNP had seven hydrogen bonds with MMP-1, particularly formed hydrogen bonds with Leu 235 and Tyr 237 residues of MMP-1. Results suggested that WNLNP could effectively interact with the active sites of MMP-1 to inhibit enzymatic activity in degeneration of ECM. Thus, except down regulating the expressions of NF-κB and MMP-1by scavenging ROS, docking result may suggested that WNLNP could also exert its antiphotoaging effect via directly inhibiting the activity of NF-κB and MMP-1.

**Figure 10:**
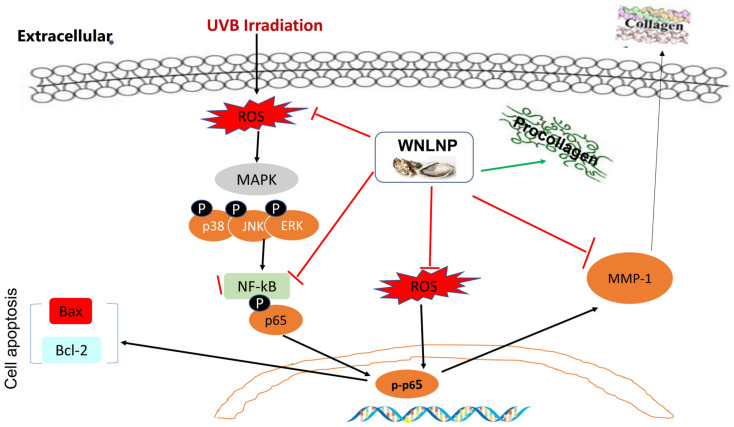
Possible mechanism by which WNLNP attenuated UVB-induced damages in HaCaT cells.

In summary, four peptides including YTVTF, RKNEVLGK, WNLNP, and VTY were separated and purified in this study. Among these four peptides, WNLNP, RKNEVLGK protected HaCat cells from damages induced by UVB irradiation. Particularly, WNLNP showed strongest MMP-1 inhibitory effect than other peptides. It may suggest that MMP-1 inhibitory peptides exert antiphotoaging effect by reducing the degradation of ECM. Further study revealed the most effective identified peptide WNLNP inhibited ROS production and MMP-1 expression, while increase the production of pro-collagen I in UVB-irradiated HaCaT cells. As important signaling pathways for the regulation of MMPs, MAPKs and NF-ΚB were activated after UVB irradiation. In addition, the expression of pro-apoptosis protein bax was promoted, while anti-apoptosis protein blc-2 expression was decreased after UVB exposure. WNLNP suppressed apoptosis in UVB-irradiated HaCaT keratinocyte cells via blocking p38, JNK, ERK phosphorylation in the MAPK signaling pathway and down-regulating NF-κB signaling pathway. Molecular mocking results suggested that WNLNP may also exert its antiphotoaging effect via directly inhibiting the activity of NF-κB and MMP-1. A schematic explanation for the UVB-mediated photoaging and the role of WNLNP has been shown in Figure 10.

Overall, this research suggested a potential role for a pentapeptide WNLNP isolated from OPH in the treatment of UVB-induced photoaging, which could be the promising ingredient in cosmetic formulations or functional foods against skin photoaging.

## 4. Materials and Methods

### 4.1. Preparation of Oyster Protein Hydrolysate with Low Molecular Weight

Hydrolysis of oyster protein was carried out according to our previous method [12], with slightly modification. Briefly, Hong Kong oysters (*Crassostrea hongkongensis*) were collected from coastal waters around Zhanjiang city, China. After homogenizing the fresh oyster meat with three volumes of double-distilled water, oyster isolation protein was prepared through the method of alkali (pH = 12) extraction and acid (pH = 4.8) precipitation. Subsequently, the freeze-dried oyster protein was reconstituted in three volumes of double-distilled water, and it was further hydrolyzed at 50 °C by neutral protease (3 × 10^4^ U/g protein, pH = 7.0) for 180 min. The enzyme in the solution was denatured by heating treatment (95 °C, 10 min) and the oyster protein enzymatic hydrolysate was further centrifuged after then. Finally, supernatants were gradually ultra-filtrated by 8 k, 5 k and 3 k Da ultrafiltration membrane (Millipore, Darmstadt, Germany) with molecular weight cutoffs. Four fractions with different molecular weight ranges including > 8 k Da, between 5–8 k Da, 3–5 k Da and <3 k Da were collected at last step. According to our previous study [12], the fraction with molecular weight below 3 k Da exerted certain photoprotective activity on an UVB-irradiated photoaging mice model. Hence, this fraction was chosen for further isolating and purifying.

### 4.2. Cell Culture & Cell Viability Test

The immortalized human skin keratinocytes cell line (HaCaT cells) was obtained from Beina Chuanglian Biotechnology (Beijing, China) Institute. Cells were cultured in Dulbecco’s modified eagle’s medium (DMEM, with high glucose) supplemented with 10% fetal bovine serum (heat-inactivated) and antibiotics (penicillin-streptomycin solution). To evaluate the antiphotoaging effects of samples, HaCaT cells were firstly planted in a 96-well plate at a density of 1 × 10^4^ cells/well. Twelve hours later, after washing cells with PBS three times and maintaining thin layer of PBS at the bottom of wells, cells were irradiated with UVB light (9 W, with wavelength ranging from 285 to 350 nm, peaked at 310–315 nm, PL-S 9W/01, Philips) at an intensity of 35 mJ /cm^2^. The ultraviolet radiation intensity of cells was measured by a UV light radiometer with UVB probe (Beijing Normal University Instrument Factory, Beijing, China). After then, the PBS was replaced with fresh medium containing samples with different treatment and cultured at 37 °C incubator with 5% CO_2_ for 24 h. Finally, cell viability of HaCaT cells was determined by Cell Counting Kit-8 (CCK-8) according to manufacturer’s instruction (DOJINDO Laboratories, Kumamoto, Japan).

### 4.3. Western Blotting

HaCaT cells suspension was seeded onto 6-well plates (1 × 10^6^/well). UVB irradiation and samples treatment were performed according to the previous Section 4.2. Twenty-four hours later, cells were lysed by cell lysis buffer (#P0013, Beyotime Biotechnology, Beijing, China) containing proteinase inhibitor PMSF. The concentrations of the protein samples were tested by BCA kit (#P0010S, Beyotime technology, Beijing, China). Expressions of MPP-1, Bax and bcl-2 and the activation of MAPK/NF-κB signaling pathway were analyzed by Western blotting assay. Briefly, 30 μg of extracted total protein samples were loaded onto 10% SDS–PAGE gel. After separating on the gels, proteins were transferred into PVDF membranes. Subsequently, membranes were blocked with blocking buffer (#P0252, Beyotime technology, Beijing, China) at room temperature for 2 h, and then incubated with antibodies of primary antibodies (Santa Cruz Biotechnology, Dallas, TX, USA, diluted 1:1000) at 4 °C overnight. Primary antibodies of MMP-1 (#sc-21731), p65 (#sc-8008), p-p65 (#sc-136548), ERK (#sc-135900), p-ERK (#sc-81492), JNK (#sc-7343), p-JNK (#sc-6254), p38 (#sc-7972), p-p38 (#sc-7973), bax (#sc-7480), bcl-2 (#sc-7382) were purchased from Santa Cruz Biotechnology, USA. After removing the first antibodies and washing with WB detergents (#sc-2005, Beyotime biotechnology, Beijing, China), PVDF membranes were incubated with secondary (Santa Cruz Biotechnology, Dallas, TX, USA diluted 1:8000) antibodies at room temperature for 2 h. Finally, PVDF membranes were detected by using enhanced chemiluminescence substrates (#F03, Wenyuange biotechnology, Nanjing, China). The image film of protein bands was photographed in a chemiluminescence imager (Tanon technology, Shanghai, China), and the bands density metrically analyzed by its supporting image analysis software. Protein bands were normalized to β-actin, and all data were expressed as X of the corresponding control groups.

### 4.4. Sephadex G-25 Purification

The enzymatic hydrolysate fraction with low molecular weight (F1) was firstly isolated using Sephacryl-G25 column (2.0 × 90 cm, 282 mL, GE Co. Ltd., Boston, MA, USA) on a protein purification system (AKTA purifier 100, GE Co. Ltd., USA). 2 mL sample (dissolved in ultrapure water, 35 mg/mL) was injected into the G25 column. Ultrapure water was also used for elution at a flow rate of 1.5 mL/min. The elute was monitored at 280 nm absorbance. 6 fractions were collected, freeze dried and further tested for evaluating their antiphotoaging effects on UVB-induced HaCaT photoaging cell model.

### 4.5. Purification by Reverse Phase Liquid Chromatography

Fraction with highest antiphotoaging activity after Sephadex G-25 purification was further separated by a C18 (5 μm, 4.6 × 250 mm), packed on reverse phase HPLC (Agilent Technologies Inc., Santa Clara, CA, USA) machine. Fractions were eluted with ultrapure water (solvent A) and a linear gradient of acetonitrile (solvent B) as the following gradients: starting from 0% solvent B to 30% in 15 min, holding at 30% solvent B from 15 to 16 min, decreasing 30% solvent B to 5% from 16 to 19 min, and maintaining at 5% solvent B from 19 to 20 min. The flow rate was 1 mL/min over 20 min, and peptides were detected at 214 nm wavelength. 6 subfractions were gathered according to its chromatogram peaks. Subfraction showing dominant peak showing antiphotoaging on UVB-irradiated HaCaT cells was collected and freeze dried for further characterization.

### 4.6. Sequence Characterization and Chemical Synthesis

The most active subfraction from HPLC was collected for sequence analysis using ultra higher performance liquid chromatography (UHPLC) (ultimate 3000, Thermo Fisher Scientific Inc., Waltham, MA, USA) packed with an electrospray ionization (ESI) source. Peptides were eluted, using 0.1% formic acid (FA)/2% ACN in water as solvent A, while 0.1% formic acid in acetonitrile as solvent B. Peptides were purified with the following gradients: starting with 6% solvent B and raised to 9%B in 8 min, to 14%B in 24 min, to 30%B in 60 min, to 40%B in 75 min, to 95%B in 3 min to elute all the peptides. The flow rate was 0.3 µL/min over 78 min. MS/MS Ionization was performed by electrospray ionization technique (ESI) in a positive ion mode using a Q Exactive™ triple quadrupole instrument (Thermo Fisher Scientific, Waltham, MA, USA). A MS/ MS full-scan was performed for each sample using acquisition with ranges of 200–1800 in MS mode, and 50–1990 in MS/MS mode. The peptide sequences were determined by analyzing and matching MS/MS data with Uniprot database. To determine the antiphotoaging activities of obtained peptides, corresponding peptides were artificially synthesized by China Peptides Co. Ltd. (Suzhou, China).

### 4.7. Evaluation of Intracellular ROS Levels in HaCat Cells

The ROS production levels were measured by fluorescence probe DCFH-DA (20, 70-dichlorodihydro fluorescein diacetate) assay kit according to its instruction (#S0033S, Beyotime Biotechnology, Beijing, China). HaCaT cells were initially planted in 24-well plates (for photographing, 1 × 10^5^ cells/well) or 96-well fluorescent labeling plates (for fluorescent informational intensity test, 1 × 10^4^ cells/well). After 12 h, cells were performed with UVB irradiation and samples treatment as described in the Section 4.2. Twenty-four hours later, culture medium was removed, and HaCaT cells were incubated with 0.2 mL serum-free medium containing DCFH-DA (10 μM) in the CO_2_ incubator for 20 min. After then, cells were washed with serum-free medium to remove the extracellular DCFH-DA. Subsequently, fluorescent images of DCF in the 24-well plate was photographed using a fluorescent inverted microscope, while fluorescent informational intensity of DCF in the 96-well plate was detected by fluorescence microplate reader.

### 4.8. Level of Extracellular Procollagen I Tested by Enzyme-Linked Immunosorbent Assay (ELISA)

HaCaT cells (1 × 10^5^ cells/well) were seeded in 24-well plates, and then performed with UVB irradiation and samples treatment as what had described in the Section 4.2. Twenty-four hours later, the cell culture supernatant was carefully gathered in tubes, and then centrifuged at 3000 rmp/min for 15 min. The supernatant was collected to measure the level of procollagen I by ELISA kits (#E-EL-H0181c, Elabscience Biotechnology, Wuhan, China), according to the manufacturer’s instruction with no change.

### 4.9. Statistical Analysis

Results were analyzed using Statistical Package for Social Sciences (SPSS, software version 17.0, IBM Co. Ltd., Almonk, NY, USA), and all data are presented as mean values ± SEM in three independent experiments. One-way analysis of variance following the LSD test was used for determination of statistical significance between groups. Significance was considered at *p* < 0.05 or *p* < 0.01.
